# A comparative analysis of smoke-free regulations in 23 Chinese cities, 2015–2022

**DOI:** 10.18332/tid/221923

**Published:** 2026-07-31

**Authors:** Yu Chen, Xinjie Zhao, Zhifeng Chen, Xinyi Zhang, Jinghan Li, Jiayi Lan, Shiyu Liu, Xinyao Yu, Kin-Sun Chan, Qian Zeng

**Affiliations:** 1 School of Art and Communication, Fujian Polytechnic Normal University, Fuqing, China; 2 School of Journalism and Communication, Peking University, Beijing, China; 3 School of Public Health, Xi’an Jiaotong University, Xi'an, China; 4 Faculty of Social Sciences, University of Macau, Macau, China; 5 Northwest University of Political Science and Law, Xi’an, China

**Keywords:** FCTC Article 8, smoke-free legislation, China, subnational law, tobacco control policy

## Abstract

**INTRODUCTION:**

China ratified the WHO Framework Convention on Tobacco Control (FCTC) in 2005 but lacks a national comprehensive smoke-free law. By 2024, 23 cities had achieved FCTC Article 8 compliance through three distinct legislative vehicles: Dedicated Tobacco Control regulations (DTC), Patriotic Health regulations (PH), and Civilization Promotion regulations (CP). This study compared their legal authority, textual scale, and FCTC-related content coverage.

**METHODS:**

Full legislative texts of all 23 FCTC-compliant city regulations, identified via the Healthy China Smoke-Free Legislation platform, were analyzed. Two coders independently extracted 16 variables (5 legislative-feature variables; 11 FCTC-related content dimensions, of which 9 were eligible for cross-vehicle comparison). Legislative scale was compared using Kruskal-Wallis H tests with exact permutation p-values and epsilon-squared effect sizes; content coverage was compared using exact chi-squared tests.

**RESULTS:**

The 23 cities protect approximately 178 million residents (12.64% of China's 2023 population), below the Healthy China 2030 target of 80%. Regulations were enacted as local statutes or government rules of differing legal rank. Legislative scale differed markedly: median clause count 27.5 (DTC) versus 3.0 (PH) and 2.0 (CP); median character count 4158 (DTC) versus 319 (PH) and 88 (CP) (both p<0.001, ε^2^>0.76). Seven of 9 FCTC-related dimensions differed significantly across vehicles - individual violator penalty, venue-operator penalty, complaint hotline, youth cigarette sales ban, tobacco advertising ban, cessation services, and health education – with DTC showing near-complete coverage and CP concentrating on Article 8 core prohibitions.

**CONCLUSIONS:**

Chinese cities implement FCTC Article 8 through legislative vehicles of differing legal rank and markedly divergent textual scope. To progress toward the 80% coverage target of Healthy China 2030, these findings support the case for comprehensive national smoke-free legislation extending protections beyond municipal-level legislative authority, ideally with graduated penalties, designated enforcement authorities, and public complaint mechanisms.

## INTRODUCTION

China is home to approximately 300 million smokers and accounts for nearly 40% of global cigarette consumption^1,2^. As a Party to the World Health Organization (WHO) Framework Convention on Tobacco Control (FCTC) since 2005, China is obligated under Article 8 to ‘adopt and implement … effective legislative, executive, administrative and/or other measures, providing for protection from exposure to tobacco smoke in indoor workplaces, public transport, indoor public places and, as appropriate, other public places’^3^. Nearly two decades after ratification, however, China has yet to enact a national comprehensive smoke-free law^1,4,5^. In the absence of national legislation, FCTC Article 8 implementation has proceeded through subnational pathways, producing a heterogeneous patchwork of municipal regulations of varying scope and legal form^6-8^.

A recent national inventory by Bi et al.^6^ identified three distinct legislative vehicles through which Chinese cities have incorporated smoke-free provisions: Dedicated Tobacco Control regulations, Patriotic Health regulations, and Civilization Promotion regulations^6^. Each vehicle draws on a different legislative tradition. Dedicated Tobacco Control regulations resemble the stand-alone smoke-free statutes pioneered internationally by Ireland’s Public Health (Tobacco) Acts and the New York Clean Indoor Air Act, in which smoke-free provisions constitute the primary regulatory purpose^9,10^. Patriotic Health regulations embed tobacco control within China’s longest-running public health mass campaign, the Patriotic Health Movement founded in 1952, which has been analyzed as a distinctive institutional mechanism linking public health, national strength, and regime legitimacy^11,12^. Civilization Promotion regulations codify behavioral norms as part of China’s ‘National Civilized City’ governance program, representing a form of expressive legislation that operates by reshaping social meaning rather than through enforcement alone^13,14^.

Despite growing English-language literature on Chinese subnational tobacco control,^6,7,15-18^, systematic textual comparison across these three legislative vehicles remains limited. Existing work has either catalogued the broader legislative landscape without in-depth comparison across vehicles^6,7^, evaluated implementation outcomes in single cities^15,16^, or analyzed determinants of policy adoption^17,18^. A fundamental question therefore merits closer examination: do the three vehicles produce equivalent regulatory content, or do they embed structurally different approaches to smoke-free compliance?

This question has direct implications for FCTC Parties worldwide that lack dedicated national tobacco control legislation and must rely on alternative legislative instruments to achieve Article 8 compliance. Indonesia, Vietnam, the Philippines, and other countries in Southeast Asia face comparable constraints^19^, and understanding whether omnibus public health legislation or expressive behavioral regulations can substitute for stand-alone smoke-free laws is a question of global policy relevance.

A systematic textual analysis was conducted of all 23 Chinese cities that had achieved FCTC Article 8 comprehensive smoke-free compliance as of December 2024, covering regulations enacted between 2015 and 2022, drawing on the Healthy China Smoke-Free Legislation platform maintained by China University of Political Science and Law. The objectives were twofold: 1) to quantify the legal authority and textual scale of the 23 regulations across the three legislative vehicles; and 2) to compare coverage of nine FCTC-related content dimensions by vehicle type.

## METHODS

### Data sources

FCTC Article 8-compliant cities were identified using the Healthy China Smoke-Free Legislation platform a digital legislative map developed in 2020 by the Health Law Research Center of China University of Political Science and Law with technical support from Caixin Data Visualization Laboratory^20^. In this study, FCTC Article 8 compliance refers to legal compliance – that is, regulations whose text establishes a 100% comprehensive indoor smoking ban without buffer periods or designated smoking rooms and with explicit enforcement authorities and penalties – rather than empirical compliance with the law on the ground. The platform classifies cities that have enacted regulations ‘most comply[ing] with Article 8 of the Convention and the implementation guidelines of Article 8’ against this textual standard. The platform has been cited as a data source by the Campaign for Tobacco-Free Kids and in peer-reviewed Chinese public health literature^21-23^.

As of December 2024, the platform listed 24 FCTC-compliant cities. Lanzhou was excluded *a priori*, before any coding was performed, on the basis of a single objective criterion defined at the study design stage: the temporal validity of the normative document underlying the city’s compliance status. The Lanzhou Implementation Rules for Public Place Smoking Control (2018), the document that had established its comprehensive smoke-free compliance, had expired at the end of its explicit five-year validity period (30 November 2023) and had not been renewed. Reverting to the parent Lanzhou Regulation on Smoking Control in Public Places (2013), which permits designated smoking areas in certain venues, Lanzhou no longer meets the FCTC Article 8 comprehensive smoke-free standard and was therefore not included. The final sample comprises 23 cities.

Full legislative texts were obtained from municipal People’s Congress websites, the National Database of Laws and Regulations, and the PKULaw database. Each regulation was classified into one of three legislative vehicle types based on its title and primary legislative purpose. The 2023 year-end resident population data were obtained from municipal statistical bulletins.

### Coding framework

A 16-variable coding instrument was developed comprising five legislative-feature variables (enacting body, approving body, implementation year, smoke-free clause count, smoke-free character count) and 11 FCTC-related content dimensions: 1) Article 8 comprehensive smoke-free status (inclusion criterion, used to define eligibility and not statistically tested); 2) explicit vaping ban; 3) individual violator penalty; 4) venue-operator penalty; 5) explicitly designated enforcement authority; 6) complaint hotline; 7) youth cigarette sales ban (FCTC Article 16); 8) youth e-cigarette sales ban (Article 16); 9) tobacco advertising ban (Article 13); and 10) cessation services provision (Article 14); and 11) health education provision (Article 12). All 11 content dimensions were coded as binary (0/1).

A tobacco control clause was operationalized as any numbered article or paragraph of the regulation that contained substantive content regulating tobacco use, smoke-free venues, enforcement, penalties, signage, vaping, advertising, cessation, education, or youth access. Cross-references that did not themselves carry regulatory content (e.g. ‘see Article X’), purely organizational headings, and the legislative preamble were not counted. Character counts were performed on Chinese characters of the substantive clause text only, using a custom Python script; punctuation, article numbers, section headings, and trailing whitespace were excluded. For Patriotic Health and Civilization Promotion regulations, only the smoke-free clauses and directly associated provisions were counted toward clause and character counts, reflecting the portion of the regulation devoted to tobacco control.

Two researchers independently coded all 23 regulations. Inter-coder agreement was high (Cohen’s κ >0.85 for all variables); disagreements were resolved through discussion and reference to the original legislative text.

### Statistical analysis

For the Civilization Promotion subgroup, pairwise textual similarity of smoke-free clauses was computed using character-level 2-gram Jaccard similarity. This metric was selected for three reasons: it captures the two-character terminological units that predominate in Chinese legal language (e.g. public places, workplaces, smoking); it is independent of document length and is therefore suitable for comparing clauses of markedly different sizes (23 to 274 characters in this sample); and it yields transparent, interpretable values bounded between 0 and 1 without requiring the construction of a document-term matrix, which would be unstable at this sample size.

Legislative scale (clause count and character count) was compared across the three vehicle types using the Kruskal-Wallis H test. Given the small size of the Patriotic Health group, exact permutation p-values (100000 resamples) were used instead of the chi-squared approximation. Effect sizes were reported as epsilon-squared (ε^2^). Coverage of nine FCTC-related content dimensions was compared across vehicles using exact chi-squared tests for 2×3 contingency tables; the Article 8 inclusion criterion was excluded as a tested dimension because it defines the sample, and the youth e-cigarette sales ban was excluded because coverage was 0% in all three groups (reflecting reliance on the 2022 national Electronic Cigarette Management Measures), which precluded meaningful cross-vehicle comparison. Population coverage was calculated as the sum of 2023 resident populations across the 23 cities and compared against the Healthy China 2030 targets (30% population protected by 2022; 80% by 2030)^24^. All analyses were conducted in Python 3.11 using SciPy 1.11. Two-sided p<0.05 was considered statistically significant. The implications of performing nine simultaneous chi-squared tests for the family-wise Type I error rate are addressed in the Limitations section.

### Ethics statement

This study analyzed publicly available legislative documents and aggregate census data and did not involve human subjects. Ethics approval was therefore not required.

## RESULTS

### Legal authority and population coverage

The vehicle distribution of the 23 included regulations was 10 Dedicated Tobacco Control regulations, three Patriotic Health regulations, and 10 Civilization Promotion regulations. All 23 regulations were enacted by formal subnational legislative bodies. Twenty-one were passed by municipal People’s Congress Standing Committees as local statutes; the remaining two (Xi’an and Qinhuangdao) were issued by municipal governments as government rules. Within the Chinese legislative hierarchy these two formats are not of equal rank: local statutes are higher in legal authority and, where their provisions conflict with government rules, prevail. Among the 21 local statutes, four enacted by municipalities directly under the central government or by provincial capitals (Beijing, Shanghai, Shenzhen, Hangzhou) did not require separate provincial-level approval; the remaining 17 received subsequent approval from provincial People’s Congress Standing Committees. Implementation dates ranged from 2015 (Beijing, Xining) to 2022 (Hangzhou, Songyuan, and several Civilization Promotion regulations). The 23 cities collectively protect approximately 178 million residents based on 2023 year-end census data, representing 12.64% of China’s 2023 year-end resident population of 1409.67 million (National Bureau of Statistics of China).

**Figure 1:**
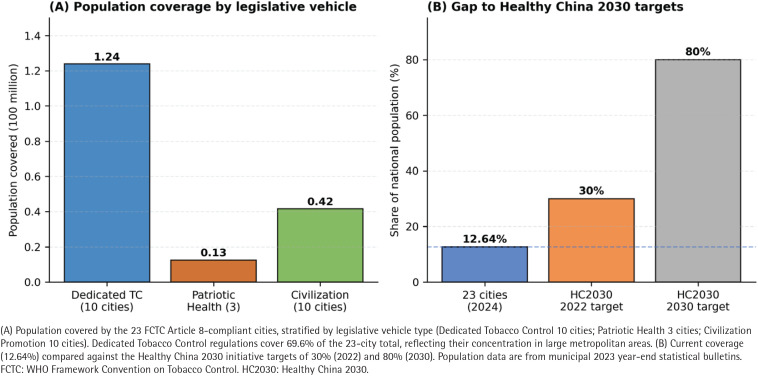
Population coverage of comprehensive smoke-free regulations across 23 FCTC Article 8-compliant Chinese cities, 2024

Population coverage was unevenly distributed across the three legislative vehicles (Figure 1A). The 10 cities with Dedicated Tobacco Control regulations collectively protect 123.9 million residents (69.6% of the 23-city total; 8.79% of national population), including all four municipalities directly under central government or special economic zones in the sample. The three Patriotic Health cities protect 12.5 million residents (7.0% of the sample; 0.89% national). The 10 Civilization Promotion cities protect 41.7 million residents (23.4% of the sample; 2.96% national). The current 12.64% coverage falls 17.4 percentage points short of the 2022 Healthy China 2030 target of 30% and 67.4 percentage points short of the 2030 target of 80% (Figure 1B)^24^.

### Legislative scale across vehicles

**Figure 2:**
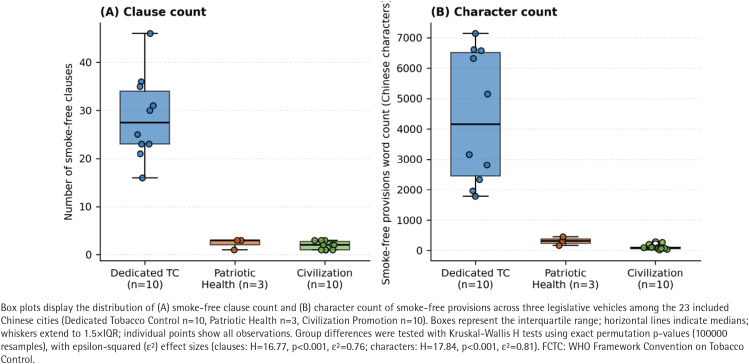
Legislative scale of smoke-free provisions by vehicle type in 23 FCTC-compliant Chinese cities, 2015–2022

Legislative scale differed markedly across the three vehicles (Table 2, Figure 2). Dedicated Tobacco Control regulations had a median of 27.5 smoke-free clauses (IQR: 16–46) and 4158 Chinese characters (IQR: 2460–6518; range 1790–7141). Patriotic Health regulations contained a median of 3 clauses (range 1–3) and 319 characters (range: 164–457). Civilization Promotion regulations had the lowest clause and character counts, with a median of 2 clauses (range: 1–3) and 88 characters (range 23–274). Kruskal-Wallis H tests with exact permutation p-values indicated statistically significant differences across vehicles for both clause count (H=16.77, p<0.001, ε^2^=0.76) and character count (H=17.84, p<0.001, ε^2^=0.81), with large effect sizes. The shortest regulation (Nanping, 23 characters) was approximately 310 times smaller in character count than the longest (Xi’an, 7141 characters).

**Table 1:** Legal authority, implementation year, and 2023 population of the 23 FCTC Article 8-compliant Chinese cities included in the study (cross-sectional textual analysis, regulations enacted 2015–2022)

*City*	*Province*	*Legislative Vehicle*	*Enacting Body*	*Year*	*Population (10k, 2023)*
Beijing	Beijing	Dedicated TC	Municipal PCSC	2015	2185.8
Shanghai	Shanghai	Dedicated TC	Municipal PCSC	2017	2487.5
Shenzhen	Guangdong	Dedicated TC	Municipal PCSC	2019	1779.0
Xi’an	Shaanxi	Dedicated TC	Municipal Government	2018	1307.8
Qingdao	Shandong	Dedicated TC	Municipal PCSC	2013	1037.2
Qinhuangdao	Hebei	Dedicated TC	Municipal Government	2019	310.7
Zhangjiakou	Hebei	Dedicated TC	Municipal PCSC	2020	405.0
Wuhan	Hubei	Dedicated TC	Municipal PCSC	2020	1377.4
Hangzhou	Zhejiang	Dedicated TC	Municipal PCSC	2022	1252.2
Xining	Qinghai	Dedicated TC	Municipal PCSC	2015	248.1
Changchun	Jilin	Patriotic Health	Municipal PCSC	2017	910.2
Yangquan	Shanxi	Patriotic Health	Municipal PCSC	2019	129.9
Songyuan	Jilin	Patriotic Health	Municipal PCSC	2022	212.6
Zhoukou	Henan	Civilization Promotion	Municipal PCSC	2021	866.6
Chenzhou	Hunan	Civilization Promotion	Municipal PCSC	2021	460.1
Xinyang	Henan	Civilization Promotion	Municipal PCSC	2020	604.8
Putian	Fujian	Civilization Promotion	Municipal PCSC	2021	317.9
Dandong	Liaoning	Civilization Promotion	Municipal PCSC	2021	209.2
Hanzhong	Shaanxi	Civilization Promotion	Municipal PCSC	2022	316.6
Nanping	Fujian	Civilization Promotion	Municipal PCSC	2022	263.0
Hebi	Henan	Civilization Promotion	Municipal PCSC	2019	156.8
Yulin	Shaanxi	Civilization Promotion	Municipal PCSC	2022	360.7
Xinxiang	Henan	Civilization Promotion	Municipal PCSC	2022	612.5
Total (23 cities)					17811.5

PCSC: People’s Congress Standing Committee. TC: Tobacco Control. FCTC: WHO Framework Convention on Tobacco Control. All 21 regulations enacted by PCSCs are local statutes; the two government-enacted regulations (Xi’an, Qinhuangdao) are municipal government rules. Within the Chinese legislative hierarchy, local statutes are higher in legal authority than government rules. Population figures are 2023 year-end resident populations from municipal statistical bulletins, expressed in units of 10000 persons.

**Table 2:** Legislative scale of smoke-free provisions across three legislative vehicles in the 23 FCTC Article 8-compliant Chinese cities, 2015–2022 (cross-sectional textual analysis)

*Metric*	*Dedicated TC (N=10)*	*Patriotic Health (N=3)*	*Civilization Promotion (N=10)*	*Kruskal-Wallis*
Clause count, median (IQR)	27.5 (23.0–34.0)	3.0 (2.0–3.0)	2.0 (1.0–2.8)	H=16.77, p<0.001, ε²=0.76
Clause count, range	16–46	1–3	1–3	
Character count, median (IQR)	4158 (2460–6518)	319 (242–388)	88 (61–102)	H=17.84, p<0.001, ε²=0.81
Character count, range	1790–7141	164–457	23–274	

TC: Tobacco Control. IQR: interquartile range. H: Kruskal-Wallis test statistic. ε²: epsilon-squared effect size. N: number of regulations in the vehicle group. Exact permutation p-values (100000 resamples) were used given the small size of the Patriotic Health group. Epsilon-squared values above 0.26 indicate large effect sizes. Character counts refer to Chinese characters of substantive smoke-free clause text only.

### Coverage of FCTC-related dimensions

**Figure 3:**
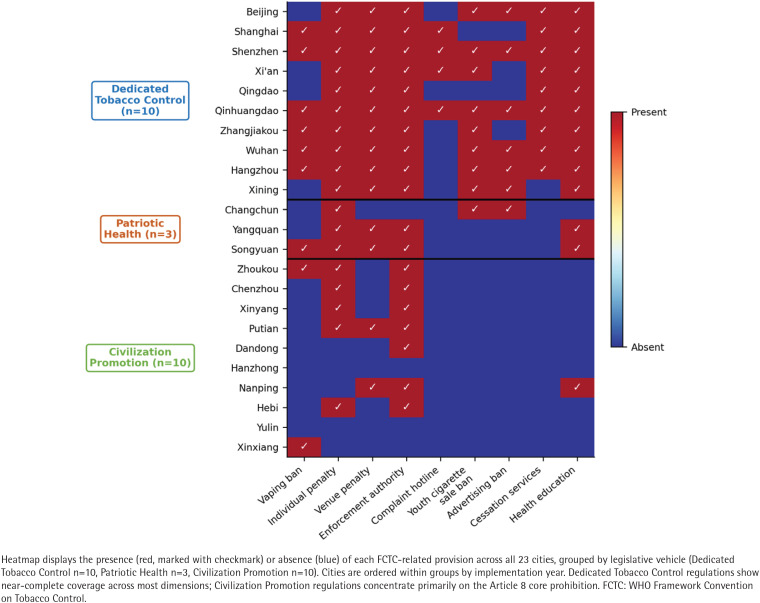
Coverage of nine FCTC-related content dimensions in 23 Chinese cities with FCTC Article 8-compliant smoke-free regulations, 2015–2022, stratified by legislative vehicle type

Coverage of the nine FCTC-related content dimensions varied substantially across vehicles (Table 3, Figure 3). Seven of the nine dimensions showed statistically significant differences in coverage at the p<0.05 level: individual violator penalty (p=0.016), venue-operator penalty (p=0.001), complaint hotline (p=0.043), youth cigarette sales ban (p=0.001), tobacco advertising ban (p=0.014), cessation services (p<0.001), and health education (p<0.001). Two dimensions did not show significant differences: explicit vaping ban (p=0.182) and explicitly designated enforcement authority (p=0.154).

**Table 3:** Coverage of nine FCTC-related content dimensions across three legislative vehicles in the 23 FCTC Article 8-compliant Chinese cities, 2015–2022 (cross-sectional textual analysis)

*FCTC-related dimension*	*DTC n (%)*	*PH n (%)*	*CP n (%)*	Exact p*
Vaping ban	6 (60)	1 (33)	2 (20)	0.182
Individual violator penalty	10 (100)	3 (100)	5 (50)	0.016
Venue-operator penalty	10 (100)	2 (67)	2 (20)	0.001
Enforcement authority	10 (100)	2 (67)	7 (70)	0.154
Complaint hotline	4 (40)	0 (0)	0 (0)	0.043
Youth cigarette sales ban (Art. 16)	8 (80)	1 (33)	0 (0)	0.001
Tobacco advertising ban (Art. 13)	6 (60)	1 (33)	0 (0)	0.014
Cessation services (Art. 14)	9 (90)	0 (0)	0 (0)	<0.001
Health education (Art. 12)	10 (100)	2 (67)	1 (10)	<0.001

DTC: Dedicated Tobacco Control. PH: Patriotic Health. CP: Civilization Promotion. FCTC: WHO Framework Convention on Tobacco Control. n: number of regulations with the dimension present. Exact chi-squared p-values were calculated for 2×3 contingency tables. The youth e-cigarette sales ban dimension is omitted because coverage was 0% in all three groups, reflecting reliance on the 2022 national Electronic Cigarette Management Measures, which precluded meaningful cross-vehicle comparison.

* p<0.05 indicate statistically significant differences across vehicles. Seven of the nine tested dimensions reached statistical significance at p<0.05 without correction for multiple comparisons; four (venue-operator penalty, youth cigarette sales ban, cessation services, health education) survive a Bonferroni-corrected threshold of α=0.0056.

Dedicated Tobacco Control regulations consistently demonstrated the most comprehensive coverage, with 100% (10/10) inclusion of individual penalties, venue penalties, enforcement authority, and health education; 90% (9/10) cessation services; 80% (8/10) youth cigarette sales bans; and 60% (6/10) tobacco advertising bans. Patriotic Health regulations showed numerically intermediate coverage on most dimensions: individual penalties 100% (3/3), venue penalties 67% (2/3), enforcement authority 67% (2/3), health education 67% (2/3), youth cigarette sales 33% (1/3), tobacco advertising 33% (1/3), and 0% (0/3) on cessation services and complaint hotlines. Civilization Promotion regulations provided the narrowest coverage, with 0% (0/10) inclusion of cessation services, youth cigarette sales bans, tobacco advertising bans, and complaint hotlines; 20% (2/10) included venue penalties; 50% (5/10) included individual penalties; 70% (7/10) included an enforcement authority; and only one (Nanping) incorporated health education.

No regulation in any vehicle explicitly prohibited the sale of e-cigarettes to minors. Explicit vaping bans appeared in 60% (6/10) of Dedicated Tobacco Control regulations, 33% (1/3) of Patriotic Health regulations, and 20% (2/10) of Civilization Promotion regulations. Complaint hotlines were included in 40% (4/10) of Dedicated Tobacco Control regulations and in none of the Patriotic Health or Civilization Promotion regulations.

### Textual similarity across Civilization Promotion regulations

**Figure 4:**
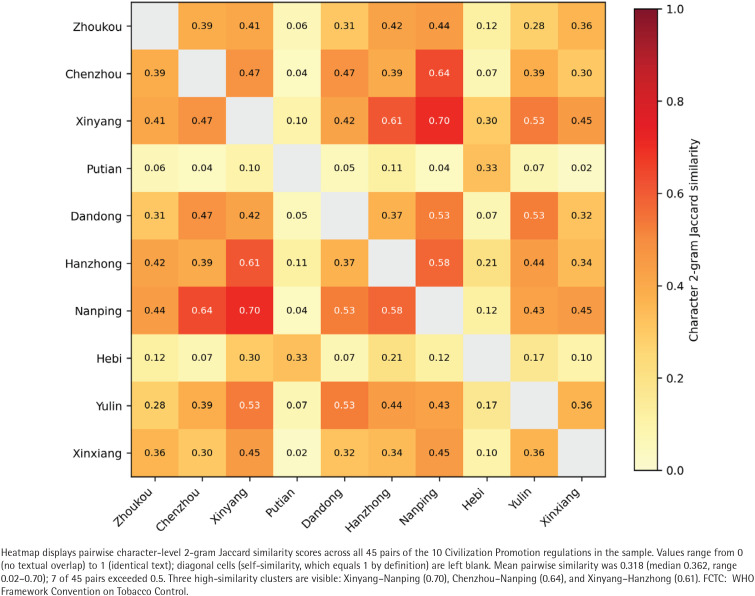
Pairwise textual similarity matrix for smoke-free provisions in the 10 Civilization Promotion regulations included in the study, 2019–2022

The 10 Civilization Promotion regulations contained smoke-free clauses ranging from 23 to 274 characters. Pairwise character-level 2-gram Jaccard similarity was computed across all 45 pairs of the 10 regulations (Figure 4). The mean pairwise similarity was 0.318 (median=0.362, range: 0.02–0.70), with 7 of 45 pairs (16%) exceeding 0.5 and 28 of 45 pairs (62%) exceeding 0.3. Three high-similarity clusters were observed: Xinyang–Nanping (0.70), Chenzhou–Nanping (0.64), and Xinyang–Hanzhong (0.61). Core linguistic elements were widely shared: 80% of regulations used the standard ‘public places, workplaces, and public transport’ triadic phrasing of FCTC Article 8, and 70% explicitly used the ‘indoor’ qualifier.

## DISCUSSION

This study offers a systematic textual comparison of the three legislative vehicles through which Chinese cities have achieved FCTC Article 8 compliance, extending the descriptive mapping provided by recent inventories^6^. Three findings warrant particular attention.

First, the 23 compliant cities cover approximately 12.64% of China’s population, well below the Healthy China 2030 targets of 30% by 2022 and 80% by 2030^24^. Even if every provincial capital and major economic center in China were to enact comparable legislation, the subnational pathway would struggle to approach the 80% target. This structural ceiling reflects an institutional feature of the Chinese legislative system: under the Legislation Law, only municipalities directly under the central government, provincial capitals, larger cities approved by the State Council, and – since the 2015 amendment – prefecture-level cities hold authority to enact local statutes, and only on enumerated matters including urban–rural construction and management, environmental protection, and historical and cultural protection. The overwhelming majority of China’s counties and county-level cities therefore lack independent legislative authority over public health matters, and this allocation of authority cannot be redrawn through municipal action. This quantitative gap lends empirical support to repeated calls – from domestic public health authorities,^1^ the Lancet Public Health^4^, and international tobacco control bodies – for comprehensive national smoke-free legislation in China.

Second, the three legislative vehicles produce fundamentally different textual architectures even when their formal legal status is comparable. Although 21 of the 23 regulations are local statutes and the remaining two are government rules of slightly lower legal rank, all confer real – if differing – enforcement authority and reach a defined municipal jurisdiction. The textual scale of smoke-free provisions, however, differs by roughly two orders of magnitude between Dedicated Tobacco Control regulations (median 4158 characters) and Civilization Promotion regulations (median 88 characters). This divergence is not arbitrary but reflects the fundamental legislative purpose of each vehicle. Dedicated Tobacco Control regulations, paralleling Ireland’s Public Health (Tobacco) Acts and the New York Clean Indoor Air Act^9^, treat smoking control as the primary legislative object and can therefore accommodate the full regulatory architecture envisioned by FCTC Article 8 and its implementation guidelines^25^: comprehensive coverage, enforcement authorities, graduated penalties, complaint mechanisms, and complementary provisions on advertising, cessation, and education. Patriotic Health regulations embed smoke-free provisions within the broader Patriotic Health Movement framework^11,12^, while Civilization Promotion regulations codify smoking restrictions alongside a long list of behavioral norms ranging from traffic courtesy to environmental cleanliness^13,14^.

Third, the variation in FCTC-related content across vehicles has direct implications for how core smoke-free provisions interact with other FCTC articles. Dedicated Tobacco Control regulations integrate provisions relevant to Articles 12 (education), 13 (advertising), 14 (cessation), and 16 (youth access) into a coherent local framework. Civilization Promotion regulations, by contrast, reduce smoke-free legislation to its declarative core: a single provision declaring that smoking in indoor public places, workplaces, and public transport constitutes uncivilized behavior. This reduction is consistent with the Sunstein^14^ theory of the expressive function of law, which holds that law can shape social norms through its declarative content independent of enforcement. Empirically, expressive legislation is thought to influence behavior by signaling collective disapproval, redefining what constitutes appropriate conduct in public space, and potentially lowering the social acceptability of smoking even where penalties are minimal or rarely applied. Whether such expressive legislation alone can sustain durable behavioral change in the absence of dedicated enforcement infrastructure remains an empirical question for future research, particularly because behavioral effects of weak-enforcement smoke-free laws have been mixed across settings^15,16,19^.

The textual similarity analysis adds nuance to the expressive-law interpretation of Civilization Promotion regulations. The moderate pairwise similarity (mean=0.318) indicates that while standard Article 8 triadic phrasing has diffused across jurisdictions as a shared regulatory vocabulary, individual cities retain expressive latitude in their specific formulations. This pattern distinguishes FCTC Article 8 diffusion from the wholesale horizontal legislative transplantation documented elsewhere in Chinese promotional local legislation, where entire regulatory structures are copied across cities^26^. The convergence observed here is more accurately characterized as linguistic standardization around a shared international norm than as domestic legislative mimicry. It is also notable that the youth e-cigarette sales ban was uniformly absent from the local texts, almost certainly reflecting reliance on the 2022 national Electronic Cigarette Management Measures, which centralized regulation of e-cigarette retail at the national level.

An important caveat applies to all three vehicles: this analysis examines the texts of formal legislation rather than what those texts achieve in practice. The literature on smoke-free policies in China consistently finds that textual coverage and behavioral coverage diverge, with implementation intensity, enforcement resourcing, public awareness, and venue-level compliance varying substantially across cities even when their formal regulations look similar^15,16,19,21^. The three vehicles studied here are likely to differ not only in what their texts say but also in the institutional infrastructure available for implementation: Dedicated Tobacco Control regulations typically come paired with a designated administrative authority and a budget line, while smoke-free provisions embedded in Patriotic Health or Civilization Promotion regulations often share enforcement responsibility with general municipal management bodies. Linking the textual architecture documented here to measured implementation outcomes is an important next step.

### Policy implications

Three policy implications follow from these findings. First, for Chinese policymakers, the persistent gap between current 12.64% population coverage and the Healthy China 2030 target of 80%, combined with the structural ceiling of subnational legislative pathways, supports the case for comprehensive national smoke-free legislation. To fulfil the FCTC Article 8 obligation of ‘effective legislative, executive, administrative and/or other measures’ protecting all persons from tobacco smoke exposure, a national law would ideally aim toward 100% smoke-free indoor public places, workplaces, and public transport with minimal exceptions, accompanied by graduated penalties for individuals and venue operators, clearly designated enforcement authorities, and functional public complaint mechanisms – elements that dedicated tobacco control regulations in our sample have demonstrated can be accommodated within a single coherent legislative instrument.

Second, for FCTC Parties that lack dedicated tobacco control legislation, our findings suggest that omnibus public health legislation or expressive behavioral regulations can achieve formal Article 8 compliance, but with a trade-off: the accompanying FCTC provisions (Articles 12–14 and 16) are substantially less likely to be integrated into such vehicles. Parties choosing these alternative pathways should anticipate that additional legislative or regulatory instruments may be required to achieve comprehensive tobacco control. Third, monitoring frameworks that classify jurisdictions as ‘compliant’ or ‘non-compliant’ with FCTC Article 8 on a binary basis may obscure meaningful variation in regulatory architecture that shapes the downstream integration of tobacco control measures into broader public health frameworks.

### Strengths and limitations

Strengths of this study include the use of a widely cited Healthy China Smoke-Free Legislation platform for case identification, double-coded analysis of all legislative texts, exact permutation p-values appropriate for small-group comparisons, and explicit linkage of findings to both Chinese policy targets and international FCTC obligations.

Several limitations should be acknowledged. First, the scope of this analysis is deliberately restricted to the textual architecture of formal legislation; enforcement intensity, behavioral outcomes, and institutional capacity – each of which varies substantially across Chinese cities regardless of legislative vehicle^15,16^ – were not assessed. The relationship between textual design and implementation effectiveness remains an important question for future research, particularly for Patriotic Health and Civilization Promotion regulations, for which rigorous implementation evaluations are currently absent from the literature. Future legislative feasibility at the national level was likewise not assessed.

Second, generalizability beyond the present sample is limited. The 23 cities are concentrated in a small set of provinces and over-represent eastern coastal and metropolitan areas; central, western, and rural-dominant jurisdictions are under-represented. Findings on textual architecture and vehicle-content patterns may therefore not extrapolate fully to the more than 600 county-level jurisdictions that have not enacted FCTC-compliant smoke-free regulations, or to regulations enacted under future legislative frameworks. Comparisons with other FCTC Parties using alternative legislative pathways – such as Indonesia, Vietnam, or the Philippines – were drawn at a conceptual rather than systematic level.

Third, the sample is restricted to cities classified as FCTC-compliant by the Healthy China Smoke-Free Legislation platform, and the analysis is therefore subject to selection bias: cities with non-compliant or partial smoke-free regulations, and cities with no smoke-free regulation at all, are not represented. The textual contrasts reported here describe the universe of cities that have achieved formal compliance; they should not be read as a description of Chinese smoke-free regulation as a whole. Conversely, by focusing on compliant cities, the study may understate the scope of textual variation present across the broader Chinese subnational legislative landscape.

Fourth, the comparison of nine FCTC-related content dimensions involved nine simultaneous exact chi-squared tests. Without correction for multiple comparisons, the family-wise Type I error rate at α=0.05 reaches approximately 37% across nine independent tests. A Bonferroni-style correction (α=0.05/9 ≈ 0.0056) would leave four dimensions – venue-operator penalty, youth cigarette sales ban, cessation services, and health education – as statistically significant. Because the cross-vehicle differences on these four dimensions are very large (typically 100% versus 0%), the substantive interpretation is unchanged, but the headline figure of seven significant dimensions should be read with this caveat.

Fifth, the Patriotic Health group contained only three regulations, which limits statistical power for detecting differences specifically involving that vehicle and means that any individual regulation in that group exerts a strong influence on group-level estimates. Sixth, the analysis focuses on static textual features rather than the dynamic amendment history of each regulation. Finally, the exclusion of Lanzhou due to the expiration of its implementation rules illustrates the fragility of compliance achieved through time-limited normative documents, but the temporal validity status of all included regulations was not systematically examined.

## CONCLUSIONS

Twenty-three Chinese cities have achieved FCTC Article 8 comprehensive smoke-free compliance through three distinct legislative vehicles whose formal legal status, while comparable in function, differs in legal rank and in textual architecture. Dedicated Tobacco Control regulations produce comprehensive local frameworks integrating multiple FCTC articles; Patriotic Health regulations embed minimal smoke-free provisions within broader public health statutes; Civilization Promotion regulations reduce compliance to its declarative core. This legislative pluralism enables formal compliance in cities without the political space for dedicated tobacco control legislation but also constrains the scope of accompanying tobacco control measures. With approximately 12.64% of the national population protected, China remains well below the Healthy China 2030 targets, underscoring the continuing need for comprehensive national smoke-free legislation to complement subnational efforts.

## Data Availability

The data supporting this research are available from the following sources: FCTC Article 8-compliant cities were identified using the Healthy China Smoke-Free Legislation platform available from https://datanews.caixin.com/interactive/2020/smokefree-digital-map.
